# Transcriptome Analysis Reveals Hub Genes Regulating Autophagy in Patients With Severe COVID-19

**DOI:** 10.3389/fgene.2022.908826

**Published:** 2022-07-18

**Authors:** Jinfeng Huang, Yimeng Wang, Yawen Zha, Xin Zeng, Wenxing Li, Meijuan Zhou

**Affiliations:** ^1^ Department of Radiation Medicine, Guangdong Provincial Key Laboratory of Tropical Disease Research, School of Public Health, Southern Medical University, Guangzhou, China; ^2^ Jiangmen Central Hospital, Affiliated Jiangmen Hospital of Sun Yat-sen University, Jiangmen, China; ^3^ Department of Radiation Oncology Ⅱ, Zhongshan People’s Hospital, Zhongshan, China; ^4^ Department of Biochemistry and Molecular Biology, School of Basic Medicine, Southern Medical University, Guangzhou, China

**Keywords:** severe COVID-19, differentially expressed genes, protein ubiquitination, RNA sequencing, peripheral blood mononuclear cells

## Abstract

**Background:** The COVID-19 pandemic has currently developed into a worldwide threat to humankind. Importantly, patients with severe COVID-19 are believed to have a higher mortality risk than those with mild conditions. However, despite the urgent need to develop novel therapeutic strategies, the biological features and pathogenic mechanisms of severe COVID-19 are poorly understood.

**Methods:** Here, peripheral blood mononuclear cells (PBMCs) from four patients with severe COVID-19, four patients with mild COVID-19, and four healthy controls were examined by RNA sequencing (RNA-Seq). We conducted gene expression analysis and Venn diagrams to detect specific differentially expressed genes (DEGs) in patients with severe disease compared with those with mild conditions. Gene Ontology (GO) enrichment analysis was performed to identify the significant biological processes, and protein–protein interaction networks were constructed to extract hub genes. These hub genes were then subjected to regulatory signatures and protein–chemical interaction analysis for certain regulatory checkpoints and identification of potent chemical agents. Finally, to demonstrate the cell type-specific expression of these genes, we performed single-cell RNA-Seq analyses using an online platform.

**Results:** A total of 144 DEGs were specifically expressed in severe COVID-19, and GO enrichment analysis revealed a significant association of these specific DEGs with autophagy. Hub genes such as *MVB12A*, *CHMP6*, *STAM*, and *VPS37B* were then found to be most significantly involved in the biological processes of autophagy at the transcriptome level. In addition, six transcription factors, including SRF, YY1, CREB1, PPARG, NFIC, and GATA2, as well as miRNAs, namely, hsa-mir-1-3p, and potent chemical agents such as copper sulfate and cobalt chloride, may cooperate in regulating the autophagy hub genes. Furthermore, classical monocytes may play a central role in severe COVID-19.

**Conclusion:** We suggest that autophagy plays a crucial role in severe COVID-19. This study might facilitate a more profound knowledge of the biological characteristics and progression of COVID-19 and the development of novel therapeutic approaches to achieve a breakthrough in the current COVID-19 pandemic.

## Introduction

The current COVID-19 pandemic, caused by novel severe acute respiratory syndrome coronavirus 2 (SARS-CoV-2), has led to urgent healthcare issues worldwide. According to the World Health Organization, 223 countries or regions had reported 456,797,217 confirmed cases of COVID-19 by 14 March 2022, including 6,043,094 deaths. The manifestations of COVID-19 vary, and most infected individuals have only mild symptoms similar to typical pneumonia or even no symptoms (Wu and McGoogan, 2020). Furthermore, mortality is mainly observed in patients with severe COVID-19 with severe respiratory failure associated with interstitial lung pneumonia and acute respiratory distress syndrome (Berlin et al., 2020). In countries that did not implement active control measures, the case fatality rate of COVID-19 was as high as ∼10% (Iype and Gulati, 2020). However, treatment options are limited to symptomatic treatment to reduce the severity of symptoms, and no curative treatment is available. Moreover, in COVID-19, especially in the severe forms, the characteristics and effects of biological reactions are still poorly understood, which prompts researchers to search for better predictors of clinical outcomes and tools to provide information for developing new therapeutic targets and appropriate therapeutic measures. Transcriptome profiling by RNA sequencing offers sufficient gene expression analysis for characterizing COVID-19 and explains biological pathways and key genes that are not yet targeted by current therapies. In this way, Mahmud et al. (2021) identified transcription factor–gene interactions, protein–drug interactions, and DEG-miRNA coregulatory networks with differentially expressed genes (DEGs) for effective treatment of COVID-19. Auwul et al. (2021) identified that common gene signatures and pathways between COVID-19 and chronic kidney disease (CKD) could be therapeutic targets in COVID-19 patients with CKD as a comorbidity using the RNA sequencing (RNA-Seq) transcriptomic dataset of peripheral blood mononuclear cells (PBMCs) infected with SARS-CoV-2.

Autophagy refers to the process of sealing a part of the cytoplasm in the double-membrane autophagosome and delivering it to the lysosome for degradation; it is an essential cellular mechanism to cope with various stress conditions (such as starvation, energy deprivation, and pathogen invasion) and maintain a steady-state balance (Feng et al., 2014). As a monitoring mechanism, autophagy is also involved in resisting the foreign invasion of viruses. In response to viral infection, the autophagic activity is activated by host cells through virus-encoded activators, cellular stresses provoked by infection, and sensing of viral constituents mediated by Toll-like receptors (TLRs) (Viret et al., 2018). As a defense mechanism during viral infection, the autophagic activity could deliver the virus or viral protein to the lysosome for degradation, transport viral nucleic acids and antigens to endolysosomal compartments for innate and adaptive immune responses, and regulate virus-induced cell death (Levine et al., 2011). SARS-CoV-2 is an enveloped, approximately 30 kb single-stranded RNA β-coronavirus (Wu et al., 2020). Several studies have demonstrated that infection with SARS-CoV-2 may be associated with autophagy. Miao et al. (2020) demonstrated that SARS-CoV-2 virus infection would block autophagy, resulting in the accumulation of autophagosomes and causing late endosomal sequestration of the homotypic fusion and protein sorting (HOPS) component VPS39. In contrast, Hui et al. (2021) provided evidence that SARS-CoV-2 promotes autophagy to suppress type I interferon response.

**FIGURE 1:**
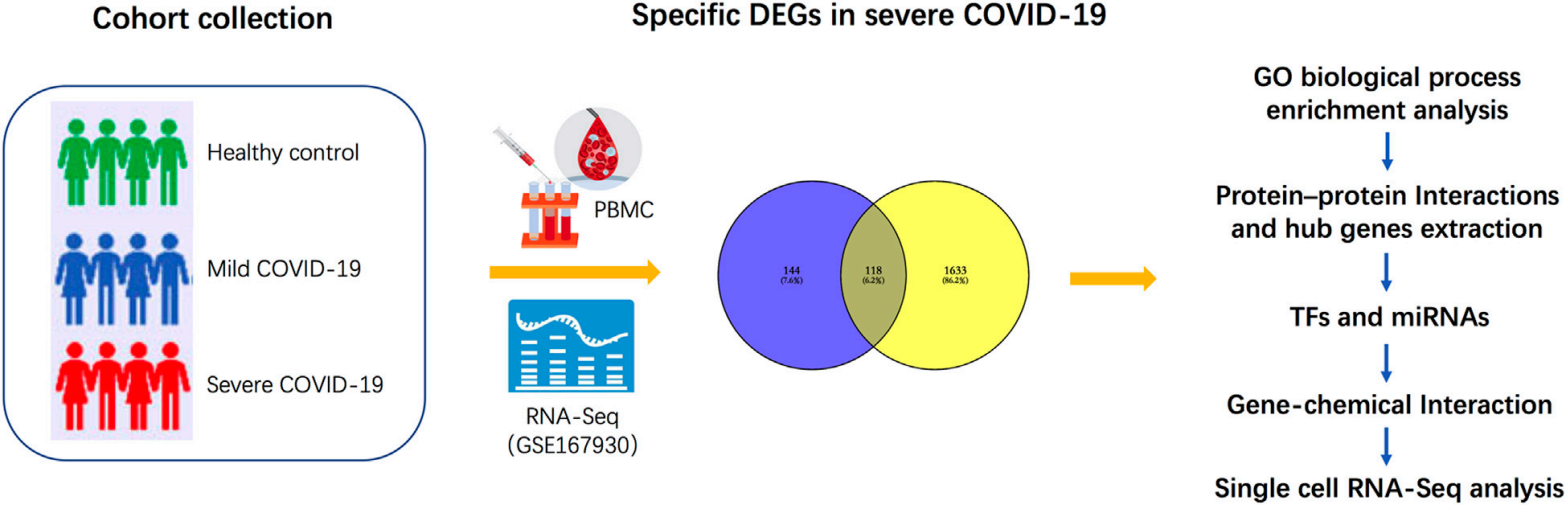
Schematic illustration of the overall general workflow of this study.

Here, to explore the biological characteristics and progression in patients with severe COVID-19 as opposed to those with mild COVID-19, we first pre-processed raw data on GSE167930 and screened specific DEGs for severe COVID-19. Gene Ontology analysis of these DEGs was performed to gain knowledge regarding their biological processes. Subsequently, we examined the most significant term and performed protein–protein interaction (PPI) network analysis to extract the hub genes regulating autophagy. Furthermore, we identified transcription factors (TFs) and microRNAs (miRNAs) at regulatory checkpoints using these hub genes. We then analyzed the protein–chemical interaction network, to determine potent chemical agents. Finally, to determine the cell type-specific expression of these genes, we performed single-cell RNA-Seq analysis using an online dataset. The sequential workflow of the processes in this study is shown in Figure 1.

## Materials and Methods

### Sample Collection, Data Processing, and Differential Expression Analysis

The study cohort comprised peripheral blood mononuclear cells from four mild COVID-19 patients, four severe COVID-19 patients, and four healthy controls. All samples were subjected to RNA-Seq analysis, and the results could be obtained from the GEO database of the National Center for Biotechnology Information (NCBI). The GEO accession ID of the dataset was GSE167930, which was already deposited for early published article by our team (Zhou et al., 2021). The limma R package was used for RNA-Seq to identify significant DEGs (the cut-off value of fold change >2 and fold change <0.5; *p*-value < 0.05). To screen specific DEGs for severe COVID-19, we set two gene clusters. In cluster 1, DEGs were significantly expressed in severe COVID-19 patients compared with healthy controls. In cluster 2, DEGs were significantly expressed in mild COVID-19 patients compared with severe COVID-19 patients. The Venn diagram of cluster 1 and cluster 2 was used to specifically distinguish DEGs associated with severe COVID-19 patients. This differential expression analysis was performed and figures were obtained using the SangerBox tools, a free online platform for data analysis (http://vip.sangerbox.com/).

### Gene Ontology Enrichment Analysis

Gene Ontology (http://geneontology.org/) stores a database of gene annotations that participate in biological processes; it calculates the probability of obtaining at least as many genes with the observed annotations. DAVID (https://david.ncifcrf.gov) was used as a data source for Gene Ontology enrichment analysis of the 144 DEGs specifically expressed in severe COVID-19, and the significant enrichments were filtered based on *p*-value < 0.05 and FDR (q-value) < 0.05. The enrichment analysis was performed, and figures mentioned earlier were obtained using the SangerBox tools (http://vip.sangerbox.com/).

### Protein–Protein Interaction Network Analysis and Hub Gene Cluster Identification

The genes involved in the crucial biological process of severe COVID-19 were included in the STRING database (https://string-db.org/) (version 11.0) to construct a PPI network. Cytoscape v.3.7.1 was then used for the visual presentation of the results from STRING. Hub gene cluster analysis was conducted using the Molecular Complex Detection (MCODE) plugin.

### Transcriptional and Post-transcriptional Network Analysis

The hub genes involved in the crucial biological processes of severe COVID-19 were used to recognize its TF gene and gene-miRNA network using the JASPAR database and TarBase database v8.0 on the NetworkAnalyst platform, respectively. Subsequently, Cytoscape v.3.7.1 was used to obtain a visual presentation of the TF gene and gene-miRNA interaction network.

### Gene–Chemical Interaction Network Analysis

The Comparative Toxicogenomics Database in the NetworkAnalyst tool was further used to recognize the relationship of potential chemical agents and hub genes in the crucial biological process of severe COVID-19. For a visual presentation of the gene–chemical interaction network, Cytoscape v.3.7.1 was used.

### Single Cell RNA-Seq Analysis

To indicate the cell type-specific expression of hub genes involved in autophagy in this study, we performed single-cell RNA-Seq analysis on a free online database platform: COVID-19 Cell Atlas Data Mining Site (http://www.covidcellatlas.com/) (Unterman et al., 2022). We set the comparison as COVID-19 Stable versus Progressive on the website, where “Stable” refers to patients hospitalized in internal medicine wards who eventually recovered and were discharged, that is, mild patients in our study, and “Progressive” refers to severe patients who required admission to the ICU and eventually succumbed to the disease. To show the identified cell types that express the gene, the UMAP Explorer was used for plotting the gene expression. We then imported the five most specifically expressed cell types into interactive connectome to explore the intercellular ligand–receptor pair interactions. All the figures were obtained from this online platform.

## Results

### Identification of Differentially Expressed Genes Specific for Severe COVID-19 Patients

**FIGURE 2:**
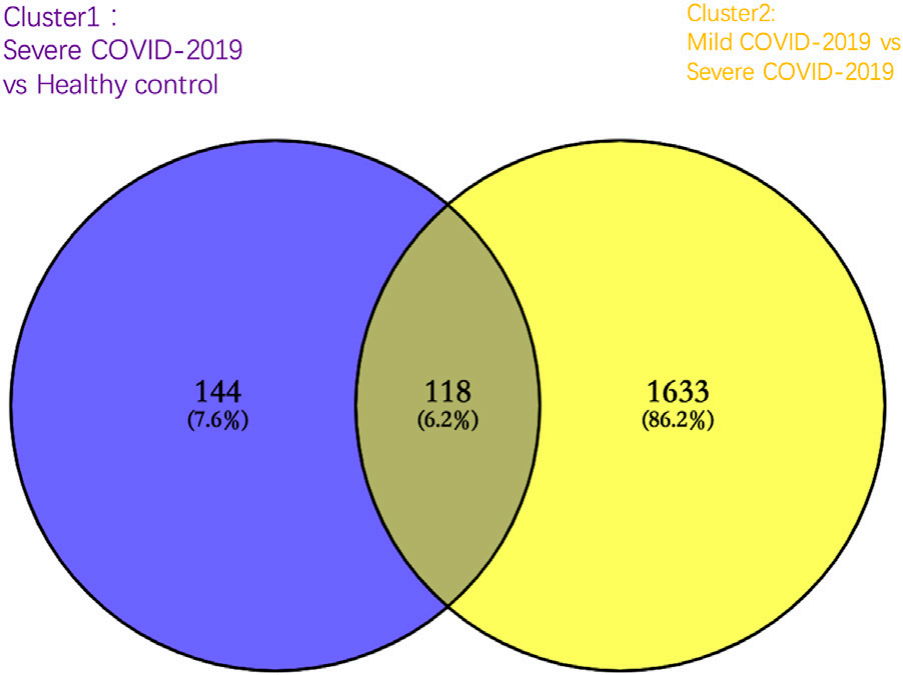
Venn diagram of significantly altered genes in cluster 1 [severe COVID-19 group compared with healthy control group (purple)] and cluster 2 [mild COVID-19 group compared with the severe COVID-19 group (yellow)]. A total of 144 genes were significantly altered specifically in severe COVID-19 patients.

To identify the DEGs specific for severe COVID-19, we first compared genes expressed in severe COVID-19 to those expressed in healthy controls and set these 262 DEGs in cluster 1 (the cut-off value of fold change >2 and fold change <0.5; *p*-value < 0.05). Following this, in cluster 2, a total of 1751 DEGs were significantly changed in mild COVID-19 patients compared with severe COVID-19 patients. As shown in the Venn diagram of cluster 1 and cluster 2, there were 144 DEGs specifically expressed in severe COVID-19, and these 144 DEGs were employed to accomplish further determination of biological process enrichment analysis (Figure 2).

### Gene Ontology Enrichment Analysis

**FIGURE 3:**
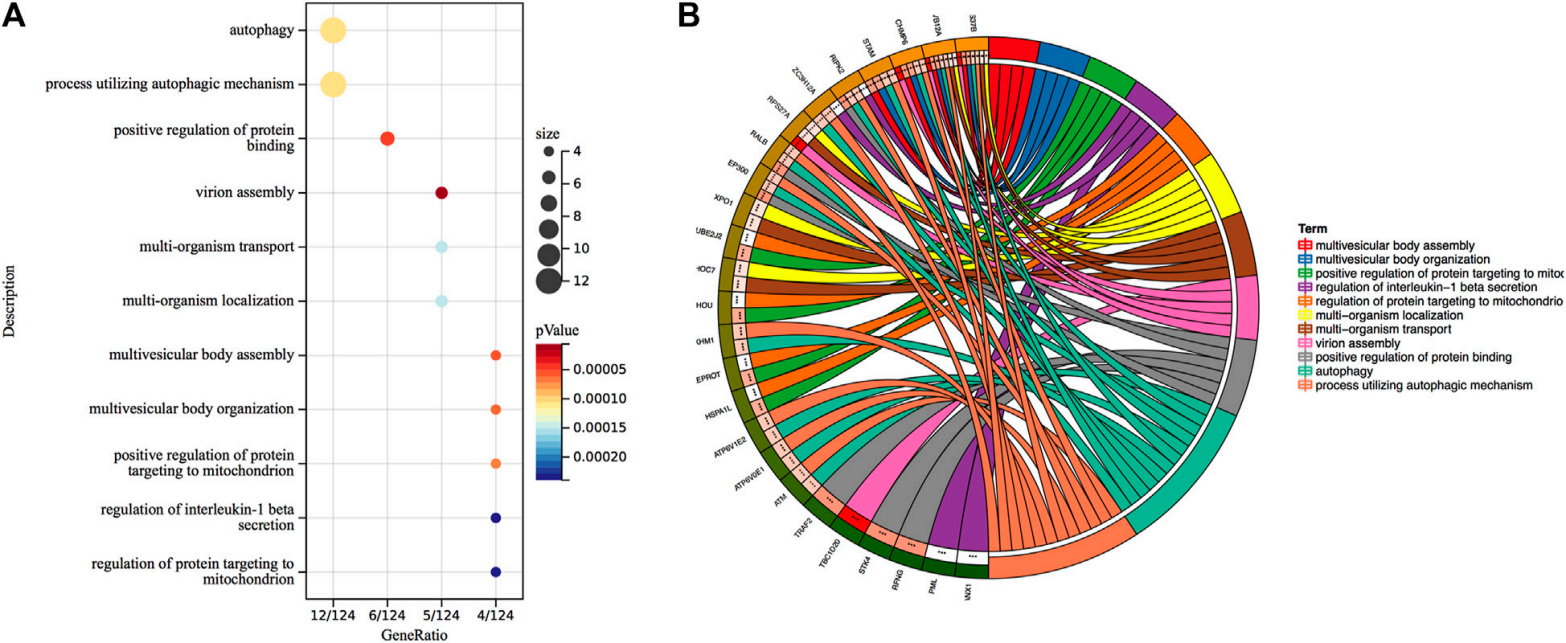
**(A)** Gene Ontology biological process enrichment analysis of 144 specific DEGs in severe COVID-19. Overall, 11 terms are presented. The size of the circle indicates the relative contribution of the genes to the activity of the term. Colors show the *p*-value. **(B)** Circles show genes contained in each term.

**FIGURE 4:**
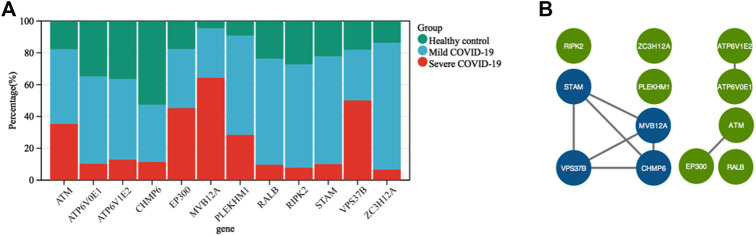
**(A)** Stacked bar chart shows the expression value percentage of the 12 genes involved in the autophagy process in patients with severe COVID-19, mild COVID-19, and healthy controls. **(B)** Protein–protein interaction network was created using STRING and visualized in Cytoscape. The core cluster analyzed by MCODE is indicated with blue color.

To gain insight into the regulation of genes and the transmission of signals that occur during the progression of severe COVID-19, we performed Gene Ontology enrichment analysis. In biological process enrichment with DAVID at *p*-value < 0.05 and FDR (q-value) < 0.05, we found a total of 11 GO terms enriched significantly for the specific 144 DEGs in severe COVID-19 patients (Figure 3). Table 1 presents the 11 GO terms based on the number of genes included. The biological process terms “Autophagy” (GO:0006914, *p*-value 0.0001, FDR 0.0327) and “Process utilizing autophagic mechanism” (GO:0006919, *p*-value 0.0001, FDR 0.0327) were considered the crucial biological process as they contained maximum 12 genes including *ATM*, *CHMP6*, *EP300*, *RIPK2*, *ATP6V0E1*, *VPS37B*, *ATP6V1E2*, *PLEKHM1*, *ZC3H12A*, *STAM*, *MVB12A,* and *RALB*. In Figure 4A, the stacked bar chart visualized these gene expressions in patients with severe COVID-19, mild COVID-19, and healthy control. Also, a total of six genes (*EP300*, *RIPK2*, *STK4*, *TRAF2*, *RFNG,* and *RALB*) were contained in “Positive regulation of protein binding” (GO:0032092, *p*-value 0.0001, FDR 0.0294). The GO term “Virion assembly” (GO:0019068, *p*-value 0.0001, FDR 0.0133) contained *TBC1D20*, *CHMP6*, *RPS27A*, *VPS37B,* and *MVB12A*. The biological process terms of “Multi-organism transport” (GO:0044766, *p*-value 0.0001, FDR 0.0365) and “Multi-organism localization” (GO:0050706, *p*-value 0.0001, FDR 0.0365) contained five genes including *THOC7*, *RPS27A*, *VPS37B*, *XPO1*, and *MVB12A*. A total of four genes (*RIPK2*, *PML*, *ZC3H12A,* and *PANX1*) were contained in “Regulation of interleukin-1 beta secretion” (GO:0050706, *p*-value 0.0002, FDR 0.0474). Then, four genes including *UBE2J2*, *LEPROT*, *RHOU,* and *HSPA1L* consist in “Regulation of protein targeting to mitochondrion” (GO:1903214, *p*-value 0.0002, FDR 0.0474) and “Positive regulation of protein targeting to mitochondrion” (GO:1903955, *p*-value 0.0001, FDR 0.0294). Furthermore, four genes (*CHMP6*, *VPS37B*, *STAM,* and *MVB12A*) were contained in terms of “Multivesicular body assembly” (GO:0036258, *p*-value 0.0001, FDR 0.0294) and “Multivesicular body organization” (GO:0036257, *p*-value 0.0001, FDR 0.0294).

**TABLE 1:** Gene Ontology biological process enrichment analysis of 144 specific DEGs in severe COVID-19 cases.

ID	Term	Size	Official Gene Symbol	*p*-value	FDR
GO:0006914	Autophagy	12	*ATM, CHMP6, EP300, RIPK2, ATP6V0E1, VPS37B, ATP6V1E2, PLEKHM1, ZC3H12A, STAM, MVB12A, RALB*	0.0001	0.0327
GO:0061919	Process utilizing the autophagic mechanism	12	*ATM, CHMP6, EP300, RIPK2, ATP6V0E1, VPS37B, ATP6V1E2, PLEKHM1, ZC3H12A, STAM, MVB12A, RALB*	0.0001	0.0327
GO:0032092	Positive regulation of protein binding	6	*EP300, RIPK2, STK4, TRAF2, RFNG, RALB*	0.0001	0.0294
GO:0019068	Virion assembly	5	*TBC1D20, CHMP6, RPS27A, VPS37B, MVB12A*	0.0001	0.0133
GO:0044766	Multi-organism transport	5	*THOC7, RPS27A, VPS37B, XPO1, MVB12A*	0.0001	0.0365
GO:1902579	Multi-organism localization	5	*THOC7, RPS27A, VPS37B, XPO1, MVB12A*	0.0001	0.0365
GO:0050706	Regulation of interleukin-1 beta secretion	4	*RIPK2, PML, ZC3H12A, PANX1*	0.0002	0.0474
GO:1903214	Regulation of protein targeting the mitochondrion	4	*UBE2J2, LEPROT, RHOU, HSPA1L*	0.0002	0.0474
GO:1903955	Positive regulation of protein targeting the mitochondrion	4	*UBE2J2, LEPROT, RHOU, HSPA1L*	0.0001	0.0294
GO:0036258	Multivesicular body assembly	4	*CHMP6, VPS37B, STAM, MVB12A*	0.0001	0.0294
GO:0036257	Multivesicular body organization	4	*CHMP6, VPS37B, STAM, MVB12A*	0.0001	0.0294

### Protein–Protein Interaction and Identification of Hub Genes Involved in Autophagy

To further acquire the core genes associated with autophagy in severe COVID-19, we conducted a PPI network of the total 12 genes involved in GO terms “Autophagy” and “Process utilizing autophagic mechanism” using STRING. In this manner, 12 nodes and eight edges were obtained with a local clustering coefficient of 0.667 and a PPI enrichment *p*-value of 0.00032 (Figure 4B). The data file was calculated by MCODE, a novel Cytoscape plugin to identify significant gene clusters. Subsequently, we obtained only one gene cluster, which consisted of four nodes (*MVB12A*, *CHMP6*, *STAM*, and *VPS37B*) and six edges. Therefore, *MVB12A*, *CHMP6*, *STAM*, and *VPS37B* were regarded as hub genes regulating autophagy in severe COVID-19.

### Determination of Regulatory Signatures

**FIGURE 5:**
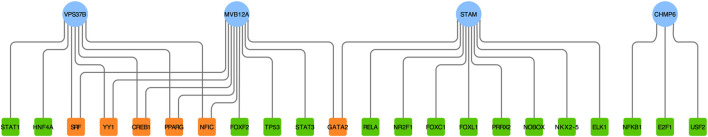
Regulatory interaction network of TF gene was identified using the NetworkAnalyst tool. Herein, the circle nodes are genes (blue); the square nodes are TFs (green); TFs targeting more than two genes simultaneously are shown in orange.

**FIGURE 6:**
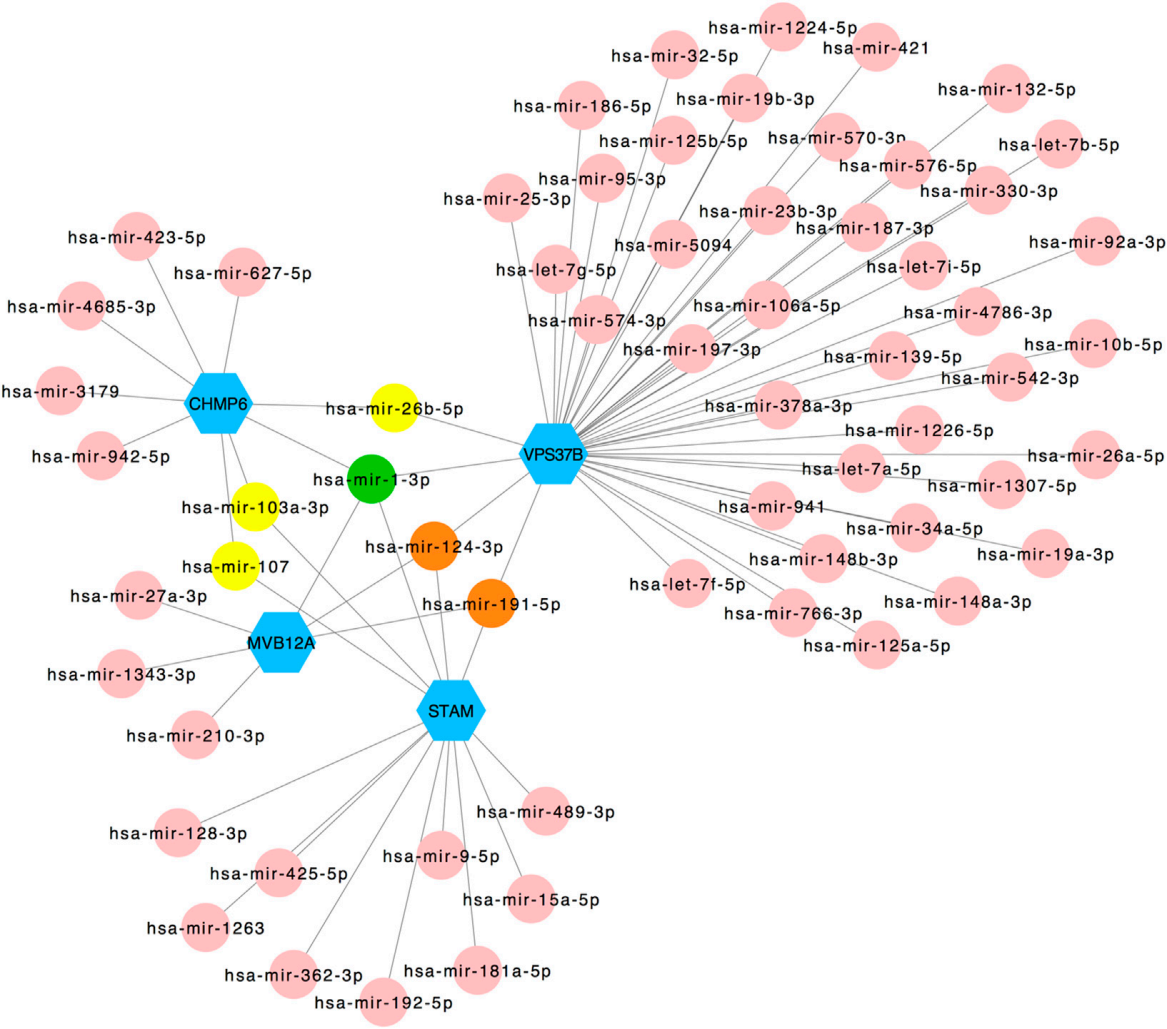
Regulatory interaction network of gene-miRNA was identified using the NetworkAnalyst tool. Genes are shown in blue; miRNAs are shown in pink; miRNAs targeting two genes are shown in yellow; miRNAs targeting three genes are shown in orange; miRNAs targeting four genes are shown in green.

The selected hub genes as mentioned earlier (*MVB12A*, *CHMP6*, *STAM*, and *VPS37B*) were evaluated with TF gene and gene-miRNA interaction network analysis to detect transcriptional signatures and post-transcriptional regulatory signatures. Figure 5 shows the TF gene interaction network drawn by Cytoscape. The transcription factors, namely, SRF, YY1, CREB1, PPARG, and NFIC, linked with *VPS37B* and *MVB12A* and GATA2 connected with *MVB12A* and *STAM*. The gene-miRNA interaction network has 66 nodes and 72 edges (Figure 6). Among these miRNAs, hsa-mir-26b-5p connected with *CHMP6* and *VPS37B*, and hsa-mir-103a-3p and hsa-mir-107 were associated with *CHMP6* and *STAM*. Subsequently, hsa-mir-124-3p and hsa-mir-191-5p were commonly linked with *VPS37B*, *CHMP6*, and *STAM*. Furthermore, we considered hsa-mir-1-3p to be the most pivotal miRNA as it was the common post-transcriptional factor for all the four hub genes.

### Construction of the Gene–Chemical Interaction Network and Identification of Potent Chemical Agents

**FIGURE 7:**
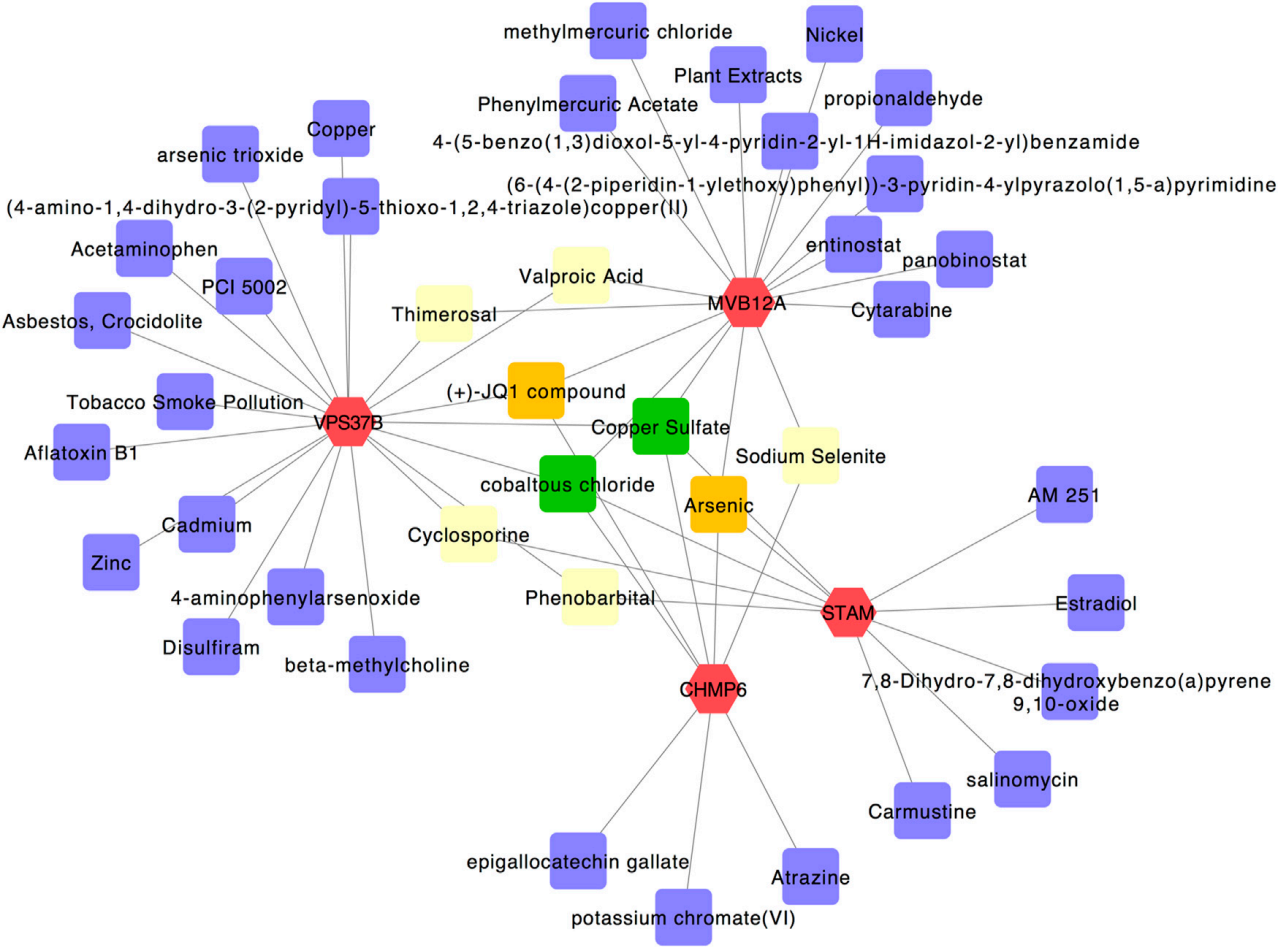
Gene–chemical interaction network. Genes are colored in red; chemical agents are colored in purple; chemical agents targeting two genes simultaneously are shown in yellow; chemical agents targeting three genes simultaneously are shown in orange; chemical agents targeting all four genes simultaneously are shown in green.

We used the four hub genes to analyze their interactions with different chemical agents in addition to using them for identifying potential antiviral agents associated with autophagy. Using the Comparative Toxicogenomics Database in the NetworkAnalyst tool, 40 chemical agents were predicted (Figure 7). Among these, thimerosal and valproic acid were linked with *VPS37B* and *MVB12A*, cyclosporine and phenobarbital were connected with *VPS37B* and *CHMP6*, and sodium selenite was associated with *MVB12A* and *CHMP6*. In addition, (+)-JQ1 compound was connected with *VPS37B*, *CHMP6*, and *MVB12A*, and arsenic was linked with *STAM*, *CHMP6*, and *MVB12A*. Most importantly, copper sulfate and cobaltous chloride were associated with all the four hub genes and could be considered to be the most relevant potent chemical agents.

### Single-Cell RNA-Seq Analysis

**FIGURE 8:**
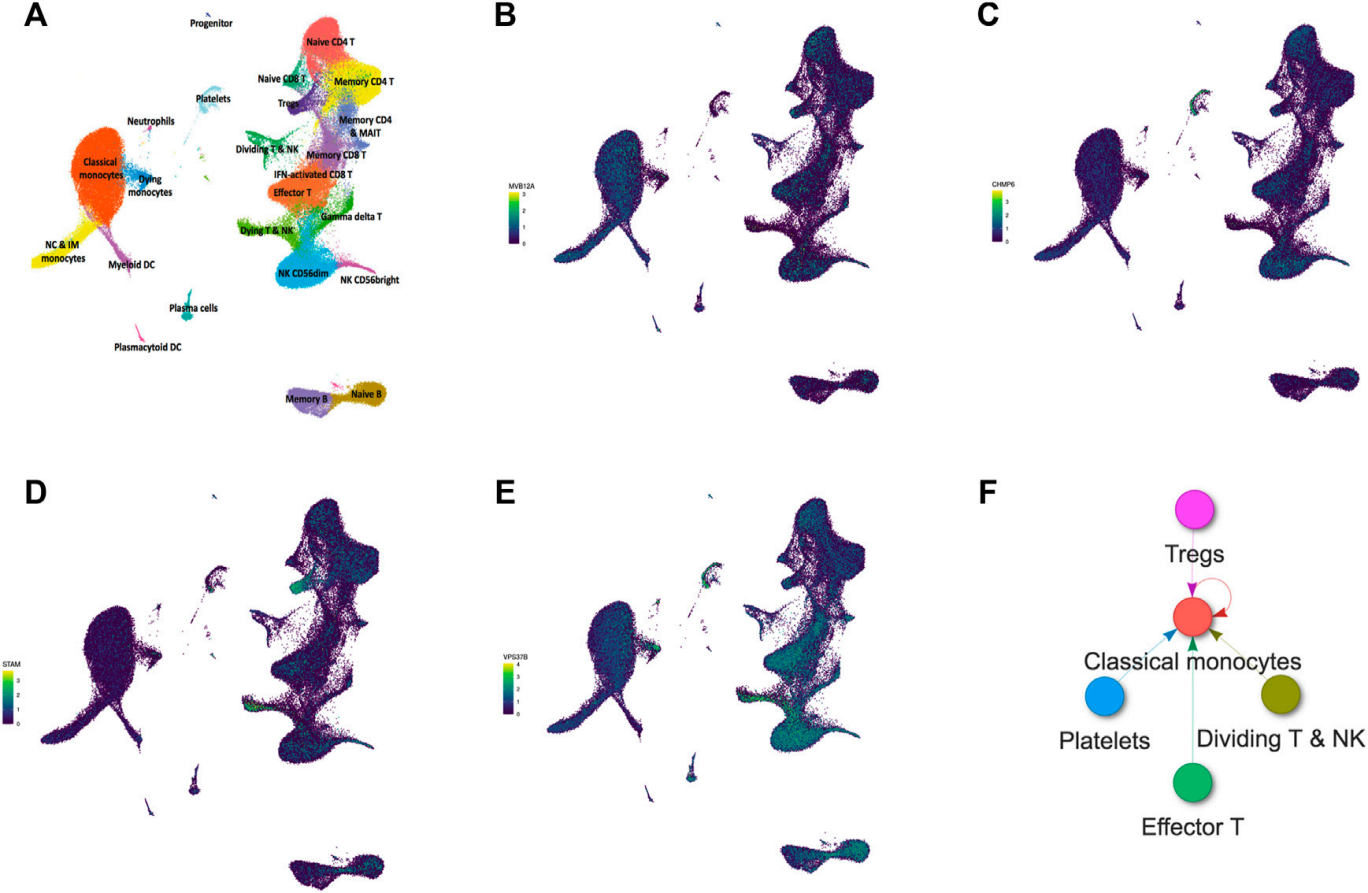
Single-cell RNA-seq analysis of PBMCs from COVID-19 patients on an online database platform. **(A)** UMAP embedding of single-cell transcriptomes from 153,554 cells color-coded for the indicated cell type. **(B**–**E)** UMAP plots showing the expressions of MVB12A, CHMP6, STAM, and VPS37B in PBMCs. **(F)** Intercellular ligand–receptor pair interactions of PBMCs from COVID-19 progressive patients.

An online single-cell RNA-Seq platform was used to assess the cell type-specific expressions of hub genes (*MVB12A*, *CHMP6*, *STAM*, and *VPS37B*). *MVB12A* was highly expressed in classical monocytes and effector T cells (Figure 8B). CHMP6 was highly enriched in plates and effector T cells (Figure 8C). *STAM* was highly expressed in Tregs and dying T & NK cells (Figure 8D). *VPS37B* was highly expressed in dying T & NK cells and effector T cells (Figure 8E). Subsequently, the interactive connectome tool in this platform was used to explore the intercellular ligand–receptor pair interactions between classical monocytes, effector T cells, plates, Tregs, and dying T & NK cells. Figure 8F shows that the ligands of effector T cells, plates, Tregs, and dying T & NK cells among PBMCs of severe COVID-19 patients are targeted to match the receptors of classical monocytes compared with the ligands of those cells of mild COVID-19 patients, and classical monocytes also secrete ligands to induce a cellular response through cognate receptors.

## Discussion

The COVID-19 pandemic is a major threat to a safe and healthy living environment of human beings and has resulted in more than 4 million deaths worldwide. It is a type of pneumonia, an infection with a virus named SARS-CoV-2 in the lungs. However, the condition is more complex. Severe COVID-19 strains display more aggressive symptoms, consequently resulting in a high mortality rate, especially the delta variant initially discovered in India in December 2020. Furthermore, the Omicron variant was discovered in South Africa a few months ago. Mild COVID-19 patients show moderate or even no symptoms. Therefore, identifying the characteristics of severe COVID-19 by comparison with mild COVID-19 would be more helpful for developing potential biomarkers and even new therapeutic targets. In this study, we first identified 144 specific DEGs in severe COVID-19 cases and performed the GO biological process enrichment analysis to acquire insight into the biological characteristics. Following this, we suggested that autophagy plays a key role in severe COVID-19, which corresponded to provide evidence in several studies. SARS-CoV-2 virus could block autophagy by infection or expression of ORF3a to sequestrate the HOPS component *VPS39* and impaired the assembly of the STX17-SNAP29-VAMP8 SNARE complex (Miao et al., 2020). Hui et al. (2021) reported that SARS-CoV-2 M protein induced mitophagy to block the downstream innate immunity signaling for inhibiting the type I IFN response. Thus, autophagy may crucially contribute to the SARS-CoV-2 viral lifecycle.

Assessment of the PPI network is considered a key pattern of protein affiliation and interaction. A total of 12 genes in the biological process of autophagy were involved in PPIs and the determination of hub genes. Here, four hub genes including *MVB12A*, *CHMP6*, *STAM*, and *VPS37B* were considered to be involved in the core regulation of autophagy in severe COVID-19 cases. Among these, Multivesicular Body Subunit 12A (*MVB12A*) is a component of the endosomal sorting required for transport I (ESCRT-I) complex. Its depletion and overexpression inhibit HIV-1 infectivity by inducing aberrant virion morphologies and altering viral Gag protein processing (Morita et al., 2007). The charged multivesicular body protein 6 (*CHMP6*) gene is the core component of endosomal sorting required for the transport III (ESCRT-III) complex, which is considered essential for viral-like particles (VLPs) and virion release (Kumar et al., 2016). The signal transducing adapter molecule (*STAM*) gene forms the endosomal sorting complex required for transport-0 (ESCRT-0), and vacuolar protein sorting-associated protein 37B (*VPS37B*) is a component of the ESCRT-I complex. All the four genes are components of the ESCRT complex, and several viruses take advantage of the ESCRT system for proliferation, budding, and transmission in infected cells (Ju et al., 2021; Meng et al., 2021). Consequently, we supposed the infection of SARS-CoV-2 may be allied to the ESCRT system.

Subsequently, the hub genes specialized for autophagy in severe COVID-19 were selected to predict their potential function at transcriptional and post-transcriptional levels. A number of transcription factors were detected. The serum response factor (SRF) has been demonstrated to modulate asymmetrical cardiac myocyte hypertrophy by constituting an epigenomic switch balancing the growth of adult ventricular myocytes in width versus length (Li et al., 2020). The transcription factor Yin-Yang 1 (YY1) played an essential role in apoptosis and angiogenesis, and its cardioprotective effects were associated with T helper 2 cytokine production and M2 macrophage polarization (Huang et al., 2021). In addition, cAMP responsive element binding protein 1 (CREB1) and its target genes identified by the recombinant canarypox vector ALVAC + Alum could augment immunogenicity and reduce the HIV-1 infection rate (Tomalka et al., 2021). Furthermore, NFIC is related to digestive system carcinoma (Fang et al., 2021) (Liang et al., 2021) and regulates renal inflammation and renal fibrosis in patients with diabetic nephropathy (Zhang et al., 2021). Eventually, hepatitis B virus x protein can interact with GATA binding protein 2 (GATA2) to influence the activity of the ST2 promoter. We detected several significant miRNAs as latent post-transcriptional factors. We believed hsa-mir-1-3p to be the most pivotal miRNA in the process of autophagy in severe COVID-19 as it was targeted by four hub genes, and hsa-mir-1-3p has been identified to have a relationship with COVID-19 in several studies (Sardar et al., 2020; Sarma et al., 2020). It can inhibit influenza A virus replication by targeting the supportive host factor ATP6V1A (Peng et al., 2018). In addition, hsa-mir-1-3p was related to tumors such as endometrial cancer, (Czerwiński et al., 2021), metastatic prostate cancer (Mukherjee and Sudandiradoss, 2021), and breast cancer (Yan et al., 2021). In addition, hsa-mir-124-3p and hsa-mir-191-5p were commonly linked with *VPS37B*, *CHMP6*, and *STAM*.

Hsa-miR-124-3p was considered a potential candidate for treating COVID-19 (Prasad et al., 2021) and regulating ACE2 networks (Wicik et al., 2020). Then, hsa-miR-191-5p showed an inhibitory effect on HIV-1 replication (Zheng et al., 2021); it is associated with cervical lesions and can serve as a non-invasive biomarker (Ning et al., 2021). Next, the chemical agents that may target the common hub genes have been detected using the Comparative Toxicogenomics Database. Among significant chemical agents, copper sulfate has been proposed as a locally applied fungicide, bactericide, and astringent in medical practice (https://go.drugbank.com/drugs/DB06778). It may induce pulmonary fibrosis through EMT activation induced by the TGF-β1/Smad pathway and MAPK pathways (Guo et al., 2021). Moreover, copper sulfate has also been identified as a potential chemical agent in pathogenetic profiling of COVID-19 (Nain et al., 2021). Furthermore, cobaltous chloride is a chemical agent that has been found to have an application in certain insecticides and fungicides (https://www.britannica.com/science/cobaltous-chloride). As the evidence of potent chemical agents in severe COVID-19 is indirective, their roles need to be further studied to be confirmed. However, although these critical factors lack experimental verification, the correlations to autophagy suggest that they play a role in the prognosis of severe COVID-19.

Eventually, for determining whether the localization of these genes regulates autophagy, we assessed the cell type-specific expressions of *MVB12A*, *CHMP6*, *STAM*, and *VPS37B* using an online single cell RNA-Seq database platform. The results showed that classical monocytes, effector T cells, plates, Tregs, and dying T & NK cells play roles in autophagy. Furthermore, classical monocytes exhibit a central role among the five cell types that constitute cellular communication because all ligands match their receptors. Monocytes are phagocytic innate immune cells in blood circulation and depending on their respective expressions of CD14 and CD16 are traditionally divided into classical monocytes (CD14^++^CD16^−^), non-classical monocytes (CD14^+^CD16^++^), and intermediate monocytes (CD14^++^CD16^+^). In acute patients with severe COVID-19, the number of non-classical and intermediate monocytes is found to be significantly reduced, whereas circulating classical monocytes display clear signs of activation (Knoll et al., 2021). Vanderbeke et al. (2021)have demonstrated that classical pro-inflammatory monocytes (based on the expressions of S100A8, S100A9, and S100A12 markers) dominate COVID-19 immunopathology in most critical cases. The results also indicated that classical monocytes were the primary source of major COVID-19-mediating cytokines, including the monocyte chemoattractant CCL2 and its receptor CCR2, the neutrophil chemoattractant CXCL8, and TNF-α (Vanderbeke et al., 2021). In addition, the expression level of the monocyte chemoattractant CCR2, which is a classical monocyte, was higher than that of non-classical monocytes, and anti-CCR2 treatment improved the course of the disease in preclinical trials (Channappanavar et al., 2016). Thus, these results demonstrate a correlation between classical monocytes and COVID-19, which could contribute to the design of novel therapeutics for this pandemic. However, because the samples used in this experiment were already used before, our conclusions may be limited by direct experimental validation. There are also few studies reported on the relationship between COVID-19 and autophagy. To the best of our knowledge, this is the first study to propose that *MVB12A*, *CHMP6*, *STAM*, and *VPS37B* are crucial genes associated with autophagy of PBMCs in patients with severe COVID-19 as opposed to those with a mild condition. Classical monocytes may play a central role in this disease; accordingly, subsequent studies should deeply explore the insight into the relationship between autophagy and classical monocytes in severe COVID-19.

## Conclusion

The present study highlights the potential specific pathogenic processes in severe COVID-19 relative to mild COVID-19 and identifies hub genes, regulatory components, and chemical agents that may help develop novel and efficacious clinical therapeutic targets. We first identified 144 specific DEGs in severe COVID-19 patients. Subsequently, using these DEGs, we identified autophagy as a critical biological process. Next, based on the PPI network, we identified the most significant gene cluster involving the hub genes of *MVB12A*, *CHMP6*, *STAM*, and *VPS37B*. Consequently, we determined that the most pivotal miRNA hsa-miR-1-3p may play a role at the regulatory level. Copper sulfate and cobaltous chloride were considered relevant potent chemical agents. Eventually, we reported that classical monocytes may play a central role in genes regulating autophagy in severe COVID-19 cases compared with mild ones. Overall, our findings will shed light on the knowledge regarding biological characteristics of severe COVID-19 cases, as well as help find novel therapeutic strategies enabling us to achieve breakthroughs in the current pandemic.

## Data Availability

The datasets presented in this study can be found in online repositories. The names of the repository/repositories and accession number(s) can be found in the article/Supplementary Material.
